# Review and Analysis of German Mobile Apps for Inflammatory Bowel Disease Management Using the Mobile Application Rating Scale: Systematic Search in App Stores and Content Analysis

**DOI:** 10.2196/31102

**Published:** 2022-05-03

**Authors:** Maximilian Gerner, Nicolas Vuillerme, Timothée Aubourg, Eva-Maria Messner, Yannik Terhorst, Verena Hörmann, Ingo Ganzleben, Hannah Schenker, Georg Schett, Raja Atreya, Markus F Neurath, Johannes Knitza, Till Orlemann

**Affiliations:** 1 Department of Medicine 1 University Hospital Erlangen, Friedrich-Alexander University Erlangen-Nuremberg Erlangen Germany; 2 Deutsches Zentrum für Immuntherapie Friedrich-Alexander-Universität Erlangen-Nürnberg and Universitätsklinikum Erlangen Erlangen Germany; 3 Autonomie, Gérontologie, E-santé, Imagerie et Société Université Grenoble Alpes Grenoble France; 4 LabCom Telecom4Health Orange Labs, Université Grenoble Alpes, Centre national de la recherche scientifique, Inria, Grenoble INP-UGA Grenoble France; 5 Institut Universitaire de France Paris France; 6 Department of Clinical Psychology and Psychotherapy Institute of Psychology and Education University of Ulm Ulm Germany; 7 Department of Internal Medicine 3 University Hospital Erlangen Friedrich-Alexander University Erlangen-Nuremberg Erlangen Germany

**Keywords:** mHealth, IBD, ulcerative colitis, Crohn disease, inflammatory bowel disease, telemedicine, mobile apps, rating, rating scale, quality, usability, chronic disease, IBS, app store

## Abstract

**Background:**

Patients suffering from inflammatory bowel disease (IBD) frequently need long-term medical treatment. Mobile apps promise to complement and improve IBD management, but so far there has been no scientific analysis of their quality.

**Objective:**

This study evaluated the quality of German mobile apps targeting IBD patients and physicians treating IBD patients using the Mobile Application Rating Scale (MARS).

**Methods:**

The German Apple App Store and Google Play Store were systematically searched to identify German IBD mobile apps for patient and physician use. MARS was used by 6 physicians (3 using Android smartphones and 3 using iPhones) to independently assess app quality. Apps were randomly assigned so that the 4 apps with the most downloads were rated by all raters and the remaining apps were rated by 1 Android and 1 iOS user.

**Results:**

In total, we identified 1764 apps in the Apple App Store and Google Play Store. After removing apps that were not related to IBD (n=1386) or not available in German (n=317), 61 apps remained. After removing duplicates (n=3) and apps for congresses (n=7), journals (n=4), and clinical studies (n=6), as well as excluding apps that were available in only 1 of the 2 app stores (n=20) and apps that could only be used with an additional device (n=7), we included a total of 14 apps. The app “CED Dokumentation und Tipps” had the highest overall median MARS score at 4.11/5. On the whole, the median MARS scores of the 14 apps ranged between 2.38/5 and 4.11/5. As there was no significant difference between iPhone and Android raters, we used the Wilcoxon comparison test to calculate *P* values.

**Conclusions:**

The MARS ratings showed that the quality of German IBD apps varied. We also discovered a discrepancy between app store ratings and MARS ratings, highlighting the difficulty of assessing perceived app quality. Despite promising results from international studies, there is little evidence for the clinical benefits of German IBD apps. Clinical studies and patient inclusion in the app development process are needed to effectively implement mobile apps in routine care.

## Introduction

In the era of COVID-19, telemedicine has become an indispensable cornerstone in the effort to maintain care of patients with chronic diseases [1-5]. Immunosuppressed patients are a fragile population, prone to infections in general, especially if they use corticosteroids [6-9]. Avoiding unnecessary face-to-face hospital visits is essential to lower the risk of infection. Remote monitoring tools, such as mobile apps [10] and video consultations [3,11] enable patient-physician communication even during the pandemic.

As inflammatory bowel disease (IBD) often affects younger people [12] who grew up interacting with mobile apps (ie, digital natives), IBD apps represent a great opportunity to improve the management of IBD patients [13]. In most cases, IBD requires life-long treatment and monitoring. One of the main goals of therapy is the prevention of disease relapses once remission has been achieved. Tight monitoring of clinical symptoms is key to ensure an adequate level of immunosuppression, control of disease activity, and quality of life. Hence, it is essential to monitor symptoms such as stool frequency, stool consistency, urgency, rectal bleeding, abdominal pain, and extraintestinal symptoms to identify disease relapses as early as possible [14]. Telemonitoring via mobile apps allows more patient-related data to be collected continuously and on demand to individually adapt therapy to each patient. Furthermore, this data can be used to generate insights into treatment efficiency, side effects, and the detailed progression of the disease.

An increasing body of evidence supports the use of mobile apps in IBD, as in other chronic diseases [15,16], to increase quality of life and medication adherence, to improve patient outcomes, and to decrease health care costs in chronic diseases such as type 2 diabetes [17], chronic obstructive pulmonary disease [18,19], and chronic heart failure [20,21]. In IBD, mobile health (mHealth) interventions to monitor patients have been shown to reduce health care visits by 33% and reduce hospital admissions without increasing disease activity or decreasing patient satisfaction [22]. In November 2019, the German government passed a law, the “Digitale-Versorgung-Gesetz DVG,” that allows a consulting physician to prescribe apps, similar to prescriptions for medical devices and drugs [23]. In order for an app to become permanently eligible for prescription via the law, a company needs to provide supporting scientific evidence. This evidence has not been provided for IBD apps that are freely available in app stores. We therefore believe it is crucial to assess the quality of these freely available IBD-related apps to adequately inform potential users.

Accordingly, the aim of this study was to review current publicly available German IBD apps for patients and physicians and rate their quality using the Mobile Application Rating Scale (MARS). MARS was developed in 2015 to objectively assess mHealth apps. It has 5 main sections (with subitems), including engagement, functionality, aesthetics, information quality, and a subjective section [24]. MARS has been used to evaluate several types of eHealth apps, such as apps for rheumatology [25], food allergies and intolerances [26], management of low back pain [27], depression self-management [28], and pain management [29].

## Methods

### Selection of Mobile Apps

We identified available apps with an extensive search in the German Apple App Store and Google Play Store in April 2020. The search included the following keywords: “Morbus Crohn,” “Colitis ulcerosa,” “CED,” “Chronisch entzündliche Darmerkankungen,” “IBD,” “Crohn’s disease,” “ulcerative colitis,” “UC,” “inflammatory bowel disease,” “Crohn,” and “colitis.” The search was carried out semiautomatically, initially using a web crawler to retrieve available apps. The app store descriptions for the available apps were read by 2 raters (JK and MG), who then manually screened them for the inclusion and exclusion criteria. The screened apps did not have to be IBD specific, but had to at least be health specific. For example, they had to include functions such as medication reminders, toilet finders, or symptom diaries. Disease-specific apps that targeted other diseases were not considered to fit the inclusion criteria. Apps were included if they were (1) in the German language, (2) available in both app stores, (3) targeted patients or physicians, and (4) were clearly designed for IBD treatment, were relevant to IBD, or were at least relevant to health in general. Apps were excluded if they were (1) only usable with an additional device, (2) congress apps, (3) journal apps, (4) apps only available to study participants or physicians, or (5) inactive apps.

### App Evaluation

All 6 raters were physicians completing their internal medicine fellowships. Half the raters (n=3) used iPhones and the other half (n=3) used Android smartphones. As recommended by the developers of MARS, all participating raters viewed the training video by Stoyanov et al [24] before rating the apps, and the raters tested each app for at least 10 minutes. The different MARS rating aspects were discussed by the team in advance. The selected apps were downloaded and rated between July and October 2020. All the raters (n=6) tested 4 of the final 14 apps (with most downloaded from the app stores), and the remaining 10 apps were randomly allocated, so that each remaining app was rated by 1 iPhone and 1 Android user.

### Statistical Analysis, Additional App Functions and App Store Ratings

Statistical analysis was implemented following the same design as Knitza et al [25], who recently performed an analysis of German mobile apps for rheumatology. MARS section scores were calculated by taking the arithmetic mean of the score for each item in a section, with the overall score being the arithmetic mean of the section scores (excluding the subjective quality score). Overall scores and section scores were summarized as the median and range for each app, and apps were ranked based on the median overall MARS score. We analyzed item score deviations by section and rater using a random intercept–only mixed-effects linear regression model including the individual item scores as the dependent variable, a random effects term for the rater, and nested random effects terms for the MARS section and app. Using random intercepts from this model, we estimated how the item scores in each section for each app deviated from the overall mean item score to rank and plot the importance of the sections within each app. Similarly, we plotted the random effect intercepts and respective 95% CIs for the raters to rank the raters by their deviation from the overall mean item score as a measure of rater bias. Random intercept and fixed effect term CIs spanning both sides of 0 were considered insignificant. Finally, we analyzed interrater agreement at the item, section, and overall score levels for raters from a rater sample as the ICC2k (2-way random, average measures, absolute agreement). All data analysis was performed using the open-source R software package (v 3.5.3; R Foundation). Mixed-effect analysis was carried out using the lme4 R package.

Additional app functions and information are shown in Table 1. The result section was generated by manually screening the final apps, checking the home page of the developers of the apps, and reading the descriptions of the apps in the 2 different app stores. This search was performed by 1 of the 6 raters (MG). The following information was systematically assessed: target group, target disease, developer of the app, app category and technical aspects, studies available, medical product, and privacy policy. For the screening of available studies, we additionally searched PubMed and Google Scholar for the app names. MG manually collected the app store ratings and the number of ratings from both app stores on Aug 24, 2021.

**Table 1:** Characteristics of the included IBD^a^ apps.

App name	Target group	Target disease	Developer	Category and technical aspects	Studies available	Medical product	Privacy policy available
De Diagnose	Patients	Nonspecific	Progressive Programming	Diagnostic support, video, audio files	No	No	Yes
Symptomate	Patients	Nonspecific	Infermedica	Diagnostic support	No^b^	Yes	Yes
Deutsches Gesundheitsportal	Patients	Nonspecific	HealthCom GmbH	Education, scientific articles, SmPC^c^	No	No	Yes
Gesina	Patients	Nonspecific	GesundHeits GmbH Deutschland	Diary, education, video call, toilet finder, video files	No	Yes	Yes
Foody	Patients	Nonspecific	Martin Stemmle, independent developer	Diary, report function	No	No	No
Carenitiy	Patients	Nonspecific	Carenitiy, Else Care SAS	Social network, education, video files, nutrition recommendation	No	No	Yes
Stuhlgang Protokoll	Patients	Nonspecific	digitalsirup GmbH	Diary, stool protocol, report function with statistics and charts	No	No	Yes
Das Schmerztagebuch-Pain Tracer	Patients	Nonspecific	Grünenthal GmbH	Education, reminder, pain diary, report function	No	No	Yes
Manage My Pain	Patients	Nonspecific	ManagingLife, Inc.	Diary, report function, medication reminder, password protection	Yes^d^	No	Yes
Alarm Medikamenten Einnahme/ Medisafe	Patients	Nonspecific	Medisafe Project Ltd., Medisafe Europe	Diary, reminder, report function	No	No	Yes
Mediteo	Patients	Nonspecific	Mediteo GmbH	Diary, reminder	No	Yes	Yes
CED Dokumentation und Tipps	Patients	IBD	Abbvie GmbH&Co KG	Diary, toilet finder, education, report function, medication reminder, stool protocol, password protection	No	No	Yes
CED-Forum	Patients	IBD	Cross4Channel—Gesellschaft für digitales Healthcare Marketing GmbH	Diary, education, social network, medication reminder, toilet finder	No	No	Yes
Cara Care	Patients	Intestinal disease^e^	HiDoc Technologies GmbH	Diary, education, reminder, audio files, report function, nutrition recommendations	Yes^f^	Yes	Yes

^a^IBD: inflammatory bowel disease.

^e^Intestinal disease: IBD, irritable bowel/gut syndrome, or gastroesophageal reflux disease.

^b^The developer website states that clinical studies are available, but they could not be identified using Google Scholar or PubMed.

^c^SmPC: summary of product characteristics.

^d^Manage My Pain–related studies [30-32].

^f^Cara Care–related studies [33].

## Results

### App Screening and Inclusion

**Figure 1:**
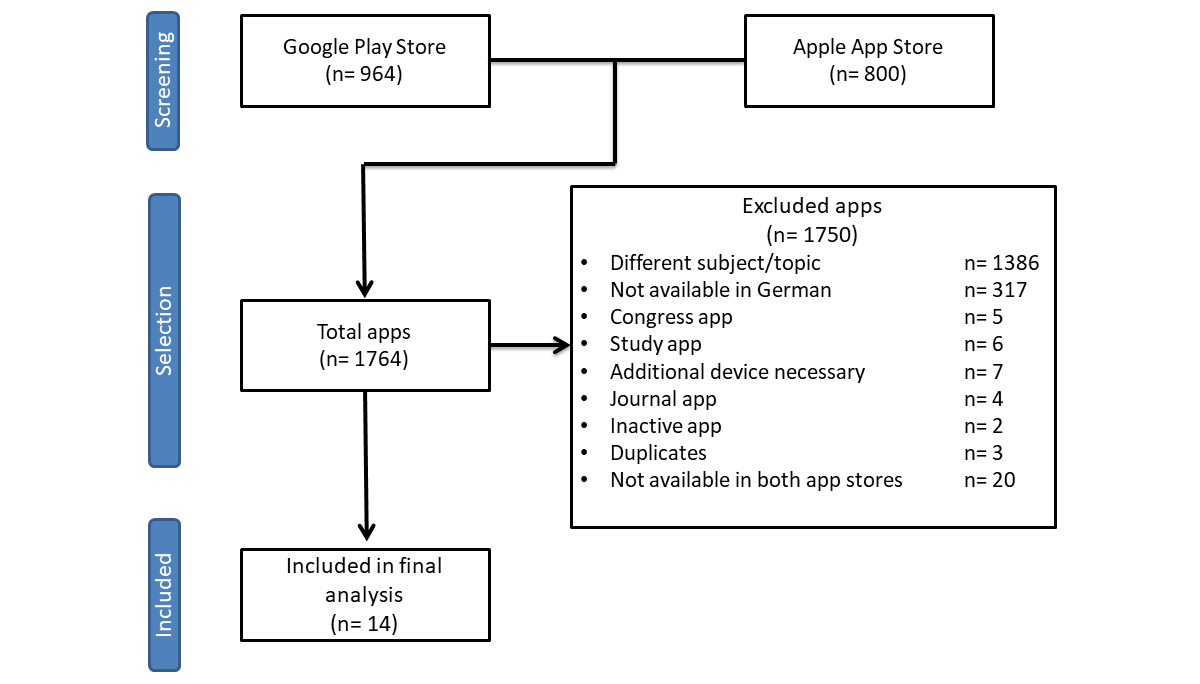
Flowchart of the app selection process.

We initially retrieved 1764 apps using the web crawler. We removed 1386 apps because they were not related to IBD, 317 apps because they were not available in German, 5 apps because they were used for congresses, and 6 clinical study apps. We also excluded 7 apps because they required an additional device, most frequently a fecal calprotectin test device. Several of these device-specific apps required an invitation for user registration or required a specific calprotectin test kit (eg, the partner apps from Abbvie, CalApp/IBDoc by Bühlmann Laboratories, and the CalproSmart by Calpro AS). The QuantOn Cal app was only usable with the specific QuantOn Cal test kit. Of the remaining apps, 20 were only available in 1 of the 2 app stores and were also excluded, as were 4 journal apps, 3 duplicates, and 1 inactive app. We included a final total of 14 apps in the MARS analysis (Figure 1).

### Characteristics of the Mobile Apps

Only 3 of the 14 rated apps (21%) addressed IBD in general, and none of them were specific to Crohn disease or ulcerative colitis. The other 11/14 apps (77%) were not IBD-specific but were relevant to IBD in other ways, such as by including pain and medication diary functions, stool protocols, or a toilet finder (see Table 1).

Importantly, we found that all of the final 14 analyzed apps addressed patients; none of them directly targeted physicians. A diary function was included in 9 of the 14 apps (64%); depending on the app, patients could track pain, frequency of defecation, or eating habits. A public toilet finder was included in 3 of the 14 apps (21%). Most of the apps had a reminder function, whether for appointments or medication. CED Forum was the only 1 of the 14 final apps that provided IBD patients an IBD-related social media platform with features similar to conventional social media platforms. It provided chatrooms on topics such as medication, symptoms, and other personal experiences, as well as diet.

Most of the apps were developed directly or indirectly by subcompanies of pharmaceutical companies. The app Carenity enabled patients to complete surveys for scientific studies; several of these can be found on PubMed or Google Scholar [34,35]. Patients did not receive compensation for completion. The app Deutsches Gesundheitsportal was the only app to directly quote research and to include articles and chapters [36]. There were also some studies based on the app Manage My Pain [30-32]. A past study analyzed patient adherence and acceptance for this app [37]. We identified 1 German study of the app Cara Care, which is for irritable bowel syndrome [33]. We classified 3 of the apps (3/14, 21%) as medical products [38-40].

### App Ratings

**Figure 2:**
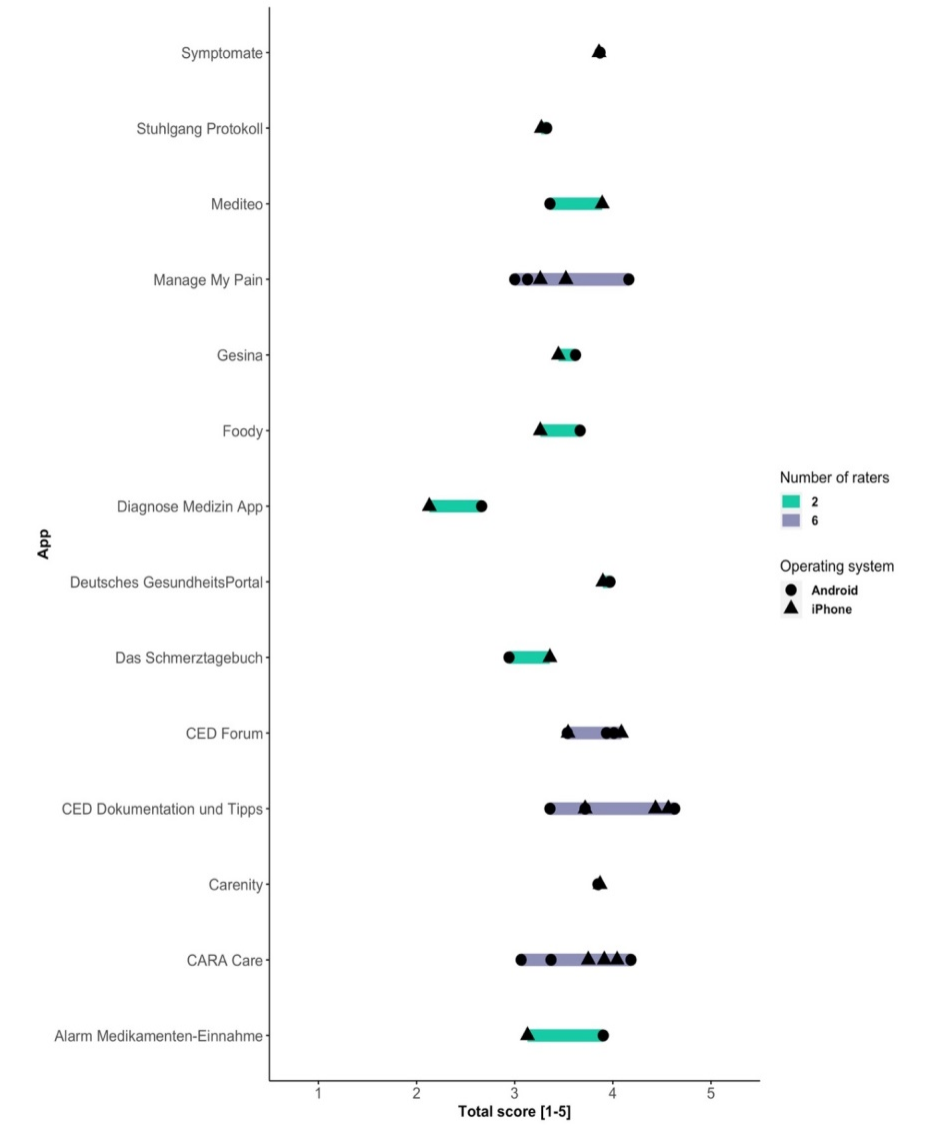
Overall MARS ratings.

Overall, app quality was varied. Median MARS scores ranged between 2.38/5 and 4.11/5. Figure 2 shows the individual MARS scores assigned by individual raters.

**Figure 3:**
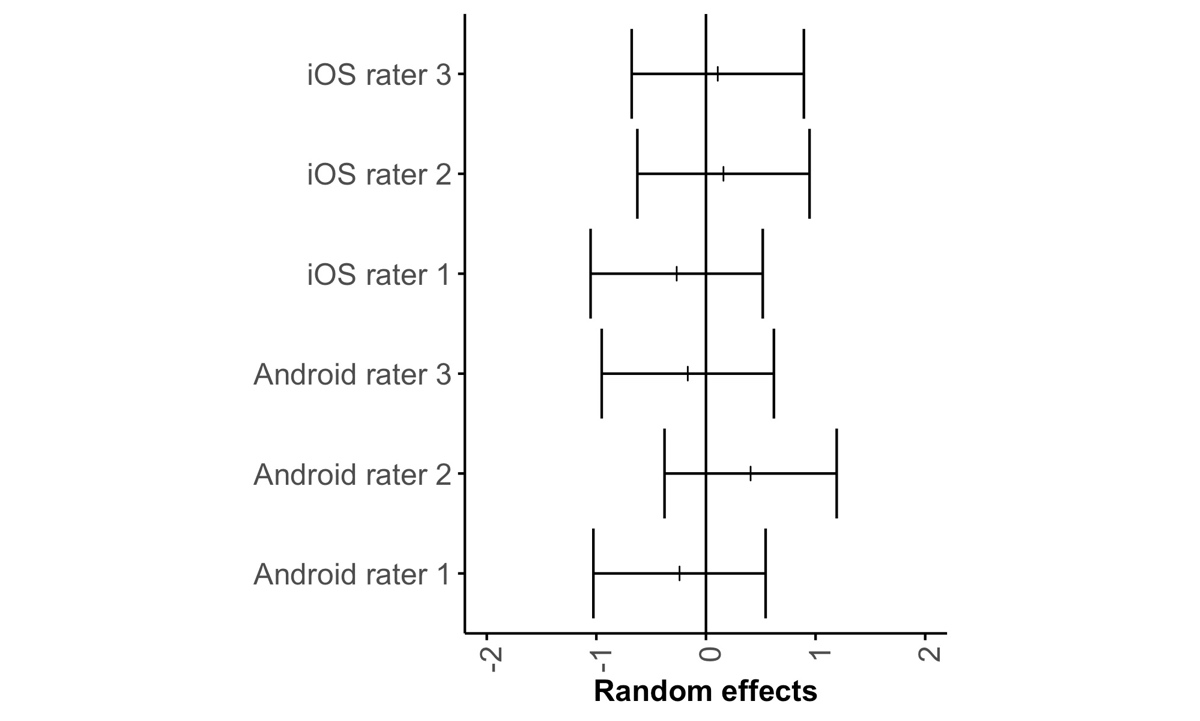
Rater deviations in item scores.

There was no significant difference between the iPhone and Android raters (*P*=.64, V=111.5). Rater agreement on the overall MARS score was good at the app level (ICC2k 0.84, 95% CI 0.68-0.93), for section score (ICC2k 0.82, 95% CI 0.76-0.88), and for individual item score (ICC2k 0.84, 95% CI 0.81-0.86). Random intercepts for observers from the mixed-effects model are presented in Figure 3.

The median total MARS and section scores are displayed in Table 2, as are the respective app store ratings and number of ratings of the respective apps. The MARS sections with the highest scores were functionality and aesthetics, with median scores of 4.12 and 4.00, respectively. Subjective quality had the lowest section score with a median of 2.88.

Multimedia Appendix 1 shows that the subjective quality section was rated systematically lower than the random intercept for each app. Otherwise, no systematic item score deviations were observed.

**Table 2:** Descriptive statistics for MARS^a^ score depending on raters and app store ratings.

App name	MARS score, median (range)				iPhone raters, n		Android raters, n		MARS section score, median (range)						Google Play Store			Apple App Store	
	Total	Android	iPhone					Aesthetics		Engagement	Functionality	Information	Subjective quality	Rating		Number of ratings	Rating		Number of ratings
Alarm Medikamenten-Einnahme	3.56 (2.98-4.14)	3.97	3.16	1		1		4.17 (3.46-4.88)		3.30 (3.16-3.44)	4.00 (3.65-4.35)	3.48 (3.03-3.93)	2.88 (1.64-4.12)	4.6		216.321	4.5		2.258
Cara Care	3.89 (3.42-4.36)	3.32 (2.71-3.93)	3.97 (3.82-4.12)	3		3		4.17 (3.45-4.89)		4.00 (3.30-4.70)	4.25 (3.62-4.88)	3.71 (3.44-3.98)	3.00 (2.18-3.82)	4.6		2.092	4.8		3.100
Carenity	3.93 (3.92-3.94)	3.94	3.93	1		1		4.67 (4.67-4.67)		3.80 (3.52-4.08)	4.12 (3.94-4.30)	3.69 (3.66-3.72)	3.38 (3.20-3.56)	4.6		134	5		1
CED Dokumentation und Tipps	4.11 (3.54-4.68)	3.79 (3.07-4.51)	4.44 (3.99-4.89)	3		3		4.50 (3.63-5.00)		4.10 (3.16-5.00)	4.75 (4.46-5.00)	4.00 (3.60-4.40)	3.62 (2.74-4.50)	3.5		118	4.3		15
CED Forum	3.97 (3.72-4.22)	3.95 (3.74-4.16)	4.07 (3.74-4.4)	3		3		4.00 (3.58-4.42)		4.40 (4.06-4.74)	4.00 (3.71-4.29)	3.76 (3.31-4.21)	3.50 (2.79-4.21)	3.8		209	3.7		30
Das Schmerztagebuch	3.17 (2.83-3.51)	2.93	3.41	1		1		3.67 (3.20-4.14)		3.10 (2.68-3.52)	4.12 (3.94-4.3)	3.21 (3.15-3.27)	1.75 (1.04-2.46)	3.7		86	3.7		15
Deutsches GesundheitsPortal	3.90 (3.88-3.92)	3.92	3.89	1		1		4.00 (3.53-4.47)		3.80 (2.67-4.93)	4.38 (3.85-4.91)	4.21 (3.44-4.98)	3.12 (2.59-3.65)	4.2		64	5		6
Diagnose Medizin App	2.38 (1.87-2.89)	2.74	2.02	1		1		2.00 (0.59-3.41)		2.40 (2.12-2.68)	3.75 (2.34-5.00)	2.50 (2.50-2.50)	1.25 (1.25-1.25)	4.5		7.179	3.8		23
Foody	3.48 (3.19-3.77)	3.68	3.27	1		1		3.33 (2.86-3.8)		3.30 (2.88-3.72)	4.12 (3.94-4.30)	3.50 (2.79-4.21)	3.12 (2.59-3.65)	4.2		225	4.5		268
Gesina	3.57 (3.53-3.61)	3.60	3.55	1		1		4.67 (4.20-5.00)		3.30 (3.16-3.44)	3.62 (3.44-3.80)	3.77 (3.16-4.38)	2.50 (2.50-2.50)	3.6		82	4.3		70
Manage My Pain	3.43 (2.97-3.89)	3.08 (2.38-3.78)	3.6 (3.4-3.8)	3		3		3.83 (3.15-4.51)		3.30 (2.71-3.89)	4.00 (3.48-4.52)	3.31 (3.05-3.57)	2.88 (2.15-3.61)	4.6		2.586	3.3		3
Mediteo	3.65 (3.29-4.01)	3.39	3.91	1		1		4.00 (4.00-4.00)		4.00 (3.72-4.28)	4.12 (3.94-4.30)	3.50 (3.03-3.97)	2.62 (1.38-3.86)	4.3		6.511	4.6		11.092
Stuhlgang Protokoll	3.35 (3.34-3.36)	3.36	3.34	1		1		3.83 (3.59-4.07)		3.20 (2.63-3.77)	4.50 (3.79-5.00)	2.97 (2.45-3.49)	2.25 (1.90-2.60)	4.4		190	4.6		31
Symptomate	3.95 (3.93-3.97)	3.94	3.97	1		1		4.67 (4.2-5.00)		3.60 (3.03-4.17)	5.00 (5.00-5.00)	3.62 (3.44-3.80)	2.88 (2.70-3.06)	4.5		3.142	4.4		8

^a^Mobile application rating scale

## Discussion

### Comparison to Previous Work

To our knowledge, no high-quality analysis of German IBD apps has yet been carried out. Our work was intended to inform patients and physicians alike about IBD apps based on the results of structured and objective testing criteria in order to guide and facilitate the selection and inclusion of appropriate IBD apps in the clinical routine.

In contrast to a previous analysis in rheumatology that used a similar search strategy [25], we did not find a single app that targeted physicians as users. This reflects an untapped potential and an opportunity, as physicians are increasingly using medical apps [41,42].

Only a few (3 of 14) of the rated apps were IBD specific: CED Dokumentation und Tipps, CED Forum, and Cara Care. None were specific to Crohn disease. Considering the relatively high incidence and prevalence of IBD [43], it was surprising that we could not identify a single disease-specific IBD app, either for Crohn disease or ulcerative colitis. In contrast, a previous analysis discovered multiple disease-specific German apps for rheumatic disease [25], including such comparatively rare types as systematic lupus erythematosus [44]. Overall, the retrieved apps addressed various patient-relevant functions and topics, but a single disease-specific app with combined app features would likely be more frequently and regularly used by IBD patients. Furthermore, such an app could include more specific topics, such as the fistulas associated with Crohn disease and IBD-associated arthritis or uveitis [45,46].

### Principal Findings

In general, information quality was rated rather poorly compared to aesthetics and functionality, representing another unmet need. As information concerning medication and disease are the top 2 features requested by patients suffering from chronic inflammatory rheumatic diseases [47], we infer that IBD patients likely also need this information. Accordingly, we are currently carrying out a patient survey to validate this assumption.

CED Dokumentation und Tipps was the app with the highest MARS score. This app was IBD specific and had several extra functions, such as a medication and appointment reminder, toilet finder, and diary function, that could be used to document pain level, stool frequency, weight, and eating habits. This app also provided nutrition recommendations, password protection, and had an especially intuitive interface and design.

Most of the apps were designed by pharmaceutical companies and did not explicitly report involving patients in their design. Notably, and in line with previous findings, very few supporting studies could be identified [25]. The app Deutsches Gesundheitsportal was the only app providing evidence (by quoting studies), while the app Carenity enabled patients to complete surveys for clinical studies. There was only one app, Manage My Pain, for which studies were available on function and patient adherence [30,32,37]. Another shortcoming of the examined apps was that none offered IBD-specific scores, such as the partial Mayo score for ulcerative colitis [48] or the Harvey-Bradshaw Index for Crohn disease [49].

In order to be eligible for prescription in Germany, developers need to provide evidence for the usefulness of their app. Only 1 of the 14 included apps, Cara Care, is expected to be among the first eligible apps in Germany related to irritable bowel syndrome and IBD. The developer homepage states that Cara Care is already eligible for prescription for patients with irritable bowel syndrome and that the developer has applied for eligibility for patients with IBD [50].

### Outlook

This study excluded apps for which a device was necessary for their use, such as the PartnerApp by Abbvie and QuantOn Cal, which both require a fecal calprotectin test device [51,52]. In our web search, we observed that these were the only devices with accompanying apps that could monitor inflammation activity in the bowel. In the future, these devices might be effective complementary apps that could provide objective data about actual disease status and other objective parameters, enabling improved remote monitoring.

Fecal calprotectin can predict relapses in IBD and indicate the response to medical treatment [53]. Furthermore, normalization of fecal calprotectin has recently been recommended as a treatment target in both ulcerative colitis and Crohn disease in the STRIDE (selecting therapeutic targets in inflammatory bowel disease) statements [54].

In several countries, including the United Kingdom and United States, IBD centers have already developed models for the use of telemedicine and have reported positive outcomes, such as decreased costs, decreased travel time, and reduced overall time for medical visits for patients [55-57]. Video consultations are used in most of these models. To the best of our knowledge, mobile apps have not been included in any of these models.

Some of the apps rated in our current study had a function allowing the creation of summary reports, for example of the last 3 months. Such reports can provide the treating physician a much more detailed and regular overview of the patient’s status, including treatment response and disease progression. The use of an additional app developed by a patient organization could be a useful supplement for improving telemedicine in IBD. Based on our study results, we suggest including the following disease-specific information in such apps: current therapy options (including evidence from major relevant clinical studies) and disease-specific scores, such as the Harvey-Bradshaw Index for Crohn disease [49] and the partial Mayo score for ulcerative colitis [48]. All major stakeholders, including patients, gastroenterologists, and scientists should be part of the app development process. In addition, studies of individual apps should be conducted to investigate their clinical and economic benefits and safety. Furthermore, the apps should be available in both app stores so that a recommendation can be made that is independent of the operating system. The developers of the apps should also be clearly identifiable. Finally, easy and secure data transmission to health care professionals should be ensured.

In the future, it may make sense to integrate the MARS score into the respective app stores in order to provide a standardized evaluation unit as an orientation aid for users. The implementation of MARS scores has been useful for the re-evaluation, optimization, and development of apps by revealing possible weaknesses of the apps and ways to improve them in a targeted manner. Evaluation with MARS should be carried out by patients and physicians as well as researchers, since future apps should ideally include 2-way communication and data exchange.

### Limitations

This study has several limitations. Importantly, the apps were all rated by physicians; no patients were included. There was a clear discrepancy between the physician MARS ratings and user ratings in the app stores, suggesting that there would also be significant differences in MARS scores if the same app was rated by a doctor and by a patient. To address this in a follow-up study, the results of this study will be discussed in a patient focus group and a reduced number of apps will be evaluated by patients. The IBD-specific apps had a significantly lower number of ratings compared to the non–disease-specific apps, which we consider was most likely due to the smaller target group. Using the web crawler, we performed an objective and automatic initial app search, similar to previous studies [58,59]. Nevertheless, some IBD apps may not have been recognized by our search strategy and might have been overlooked. Similarly, only apps available in both app stores were included. No detailed data safety analysis was performed, and we only assessed the availability of privacy policy information. Some apps also offered password protection. We excluded apps from our study that required additional devices, such as calprotectin test devices, because most of them were only accessible within specific clinical study programs and no funding was available to buy the devices. As some studies have already shown, the use of additional devices to provide objective and predictive laboratory data, such as from fecal calprotectin tests, is very useful for disease management and improves treatment [15-17,22,53,54]. The immense speed of mHealth development is also a general limitation on research in this area.

### Conclusion

Our current study shows that at the moment, only a limited number of IBD-related apps are available to patients, and none are available to physicians. We found that app quality was varied, and we observed a general absence of clinical evidence and patient involvement.

## Appendix

Multimedia Appendix 1 MARS section item scores by each section and app.

## Abbreviations

IBD: Inflammatory bowel disease
MARS: mobile application rating scale
mHealth: mobile health
