# Supplementary figures and images for: Review and Analysis of German Mobile Apps for Inflammatory Bowel Disease Management Using the Mobile Application Rating Scale: Systematic Search in App Stores and Content Analysis
Source: JMIR Mhealth Uhealth. 2022 May 3;10(5):e31102. doi: 10.2196/31102 (PMC9115651; doi:10.2196/31102)

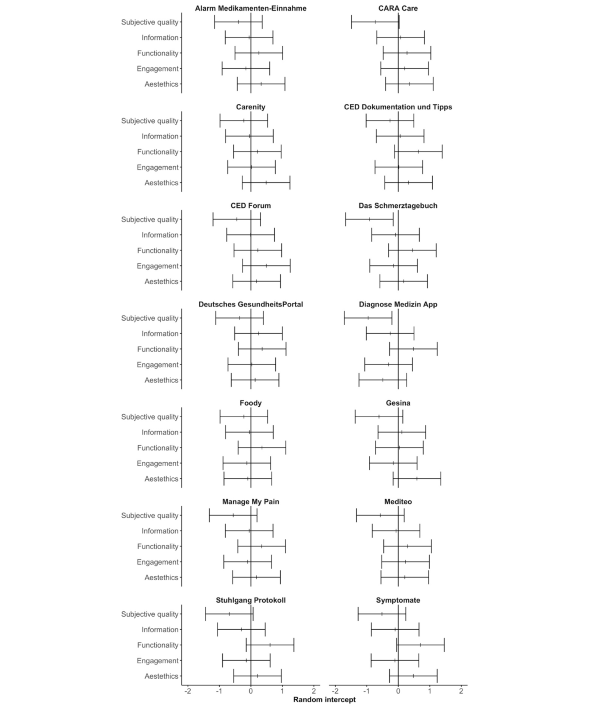

Supplement: Multimedia Appendix 1 [file mhealth_v10i5e31102_app1.png]
